# Sequential (two-step) day 3/day 5 frozen-thawed embryo transfer: does it improve the pregnancy rate of patients suffering recurrent implantation failure?

**DOI:** 10.25122/jml-2022-0041

**Published:** 2022-11

**Authors:** Soheila Arefi, Mina Ataei, Narges Maleki, Nahid Yari, Saeid Razi, Sara Amirajam

**Affiliations:** 1 Monoclonal Antibody Research Center, Avicenna Research Institute, ACECR, Tehran, Iran; 2 Department of Obstetrics and Gynecology, Social Determinants of Health Research Center, School of Medical Sciences, Alborz University of Medical Sciences, Karaj, Iran; 3 Reproductive Biotechnology Research Center, Avicenna Research Institute, ACECR, Tehran, Iran; 4 Avicenna Infertility Clinic, Avicenna Research Institute, ACECR, Tehran, Iran; 5 Bahman Infertility Center, Tehran, Iran

**Keywords:** repeated implantation failure, day 5 embryo transfer, sequential embryo transfer, randomized clinical trial

## Abstract

The best time of endometrial receptivity is the missing part of the implantation puzzle in patients with recurrent in vitro fertilization (IVF) failure. There are various treatment plans and strategies to meet the best endometrial timing for implantation. However, the lack of synchronization of the good-quality embryo with the patient's individual “window of implantation” is the hypothesis for most IVF failures so far. Sequential embryo transfer (ET) theoretically extends the availability time of embryos on the window of implantation. The study aimed to evaluate the improvement of pregnancy rate in sequential (two-step) frozen-thawed embryo transfer (FET) on day 3/day 5 in individuals who suffer from repeated IVF failures. This randomized controlled trial study was done in a university-affiliated infertility center for women with repeated consecutive IVF failures. Two hundred women aged 20–39 years who met our inclusion criteria were included in the study between January 2020 and September 2021. Participants were allocated with a 1:1 ratio to either sequential (two-step) ET on day 3/day 5 (study group, n=100) and conventional day 5 FET (n=100, control group). The frozen-thawed embryos were transferred to hormone replacement therapy-prepared endometrium in both groups. The primary outcomes were clinical pregnancy and implantation rates. The secondary outcomes were early pregnancy loss and multiple pregnancies. The demographic and clinical characteristics of the two groups were comparable. Clinical pregnancy rates were significantly higher in the sequential (two-step) FET group (40%) compared to the day 5 group (19%) (P<0.001). The sequential transfer of frozen-thawed embryos on day 3/day 5 was more effective than regular day 5 for patients suffering from repeated IVF failure.

## INTRODUCTION

Repeated implantation failure (RIF) is observed the moment embryos fail to implant after 3 attempts of transferring one to two good-quality embryos in each cycle [1, 2]. The estimated prevalence of RIF is 15%, so a considerable number of couples leave frustrated and desperate for explanations [3]. The optimal condition for a successful implantation is transferring a good-quality embryo to a receptive endometrium. Although assisted hatching, co-culture, blastocyst transfer, endometrial injury, and tailoring stimulation protocols increase *in vitro* fertilization (IVF) success rate, many infertile couples cannot attain the needed result and effective conception [4–6].

Implantation failure of endometrial origin is due to the inability to synchronize the developing embryo with the patient's implantation window (WOI) [7]. Sequential (two-step) transportation of embryos has been suggested as one of the ways to enhance the means of implantation frequency [8, 9]. Theoretically, sequential (two-step) embryo transfer (ET) extends the availability time of different-stage embryos on the window of implantation. Also, the embryo itself can induce endometrial receptivity [10], so the day 3 embryos may increase the chance of day 5 embryo implantation.

Research has been done with various designs and different results in this context. Lédée-Bataille revealed that sequential and blastocyst transfer techniques minimize the risk of cancelation and similar implantation rates [11].

In contrast, some literature did not present any considerable difference in the pregnancy rates among the two groups and those who did not have the second transfer [12–14].

Overall, preliminary evidence proposes that sequential two-step embryo transfer may have some advantages; however, careful and well-designed randomized controlled trials are essential in confirming its value in women with RIF. Available data in the field of sequential transfer are narrow and cannot be reliable. Likewise, the efficiency of this method remains a matter of discussion. Therefore, this study aimed to determine the pregnancy rate in the sequential (two-step) transfer of embryos on day 3 and day 5 compared to day 5 only in patients with at least three consecutive IVF failures.

## MATERIAL AND METHODS

The scientific and ethical board (#IR.ACECR.AVICENNA.REC.1399.001) approved this randomized clinical trial of Avicenna Research Institute (university-affiliated), recorded in the Iranian Registry of Clinical Trials (IRCT20200421047152N1). Samples were gathered from individuals referred to Avicenna Fertility Clinic, a tertiary center for recurrent pregnancy loss and infertility treatments, between January 2020 and September 2021. Females with frequent IVF/ET failures (>3 trials), 20–39 years old, with more than three good-quality frozen embryos, were recruited into the study. Two hundred ten infertile women who met our criteria were studied.

Using the following mean difference formula for two independent groups, according to a study by Balaban et al. [15], (mean 1=2.2; mean 2=2.5, SD1 and 2 were estimated by Hozo's Method as 0.5), considering α=0.05 and power of 95%, and attrition rate of 30%, 100 patients were selected for each group.


$$n=\frac{{\left(Z_{\left(1-\frac{\alpha }{2}\right)}+Z_{\left(1-\beta \right)}\right)}^{2}\left(sd_{1}{}^{2}+sd_{2}{}^{2}\right)}{d^{2}}$$

After obtaining written consent, the patients were assigned to one of the two groups through simple random samplings. Participants were assigned to one or the other sequential transfer two-step, day 3/day 5 (study group), or blastocyst embryo transportation (ET) on day 5 protocols (control group).

The inclusion criteria were normal fetus, normal screening of immunological and thrombophilia condition, lack of endometrial disorders by hysteroscopy examination and endometriosis, and presence of five embryos (having at least three embryos with good quality that can be transferred).

Patients with major uterine abnormalities and pathologies, hydrosalpinx, endometriosis, severe male factor infertility, inadequate ovarian reserve identified by AMH less than 0.5 or antral follicle count less than five, and having medical diseases were excluded from the research.

*Primary outcome:* Clinical pregnancy and implantation rates.

*Secondary outcome:* Number of oocytes retrieved, number of transferred embryos, implantation rate, twin pregnancy, and abortion rate.

### Controlled ovarian stimulation and oocyte retrieval

Patients experienced controlled ovarian stimulation by employing standard antagonist protocol until a minimum of 3 or more follicles attained a mean diameter of eighteen millimeters. Oocyte retrieval was done over 36 hours after a 250 ug injection of recombinant HCG (Ovitrelle, Merck-Serono, Switzerland).

### Intracytoplasmic sperm injection and embryo culture

After four to six hours of retrieval from the patient, an intracytoplasmic sperm injection was done. Cultures of injected oocytes were independently done using oil in 20 µl droplets of global total single-step medium (IVF online, Guelph, ON, Canada) at thirty-seven degree Celsius in an atmosphere of 6% CO_2_ and 5% O_2_. Supervision of fertilization was done sixteen to eighteen hours following injection using 2 pronuclei. Every embryo was frozen.

### Freezing-thawing procedure

Vitrification of the embryos was conducted on days 3 and 5 using the Cryotop technique [16]. The devices employed for freezing were Kitazato vitrification media and the Cryotop (Kitazato, Tokyo, Japan). On days 3 and 5, the embryos present in a similar patient were thawed on a similar day by using Kitazato thawing media, abiding by the standard protocol. The embryos were cultured independently in 20 µl droplets of global total medium under mineral oil at 37℃ in 5% CO_2_ in the air until embryo transfer following the thawing process [16].

### Embryo grading

Excellent-quality cleavage-stage (day 3) embryos are described as six to eight cells of the same size with less than ≤10% fragmentation. The blastocysts with high quality (day 5 embryos) were described as uniform blastomeres with many tightly packed cells with less fragmentation.

### Embryo transfer

The two groups were linked based on inclusion criteria, and stimulation was related to the women in the study group considering the age of delivery, the cause of infertility, ovarian stimulation protocols, the number of oocytes recovered, and the number of embryos recovered. The entire ETs were done using the Sydney IVF catheter (k-jets-7019-SIVF; Cook IVF). In the sequential D3/D5 group study group, one of the embryos was transported on day 3, and the rest remained cultured until day 5. Those embryos which had a better quality were located inside a blastocyst culture medium (Quinn's Advantage Blastocyst Medium; ORIGIO) and cultured till day 5, and one to two good-quality blastocysts were transported. In the control group, two blastocysts were transferred on day 5. The above two embryos were transported in some cases based on guideline no 182, 2006, suggested in exceptional cases with poor prognoses and RIF cycles [17].

### Luteal support and pregnancy

The luteal phase was assisted with Endometrin (Cyclogest 400; Actover Pharmaceuticals, IRAN) two times every day for a minimum of 15^th^ days following ET and persisted until nine to twelve weeks of gestation in pregnant women.

### Outcome measures

The major results for embryo transfer success included the number of the transferred embryo, rate of clinical pregnancy, miscarriage rate, and multiple pregnancy rate.

The clinical pregnancy rate was related to the primary outcome measures where chemical pregnancy was evaluated through pregnancy testing conducted 15^th^ days following embryo transfer. The definition of positive pregnancy is a B-hCG level>10 mIU/mL. Clinical pregnancy can be described as an intrauterine sac consisting of a heart pump 6 weeks following ET. Early pregnancy loss is the failure of pregnancy, which occurs before the 15^th^ week of gestation.

### Data collection and statistical analysis

Baseline characteristics like age, body mass index (BMI), the extent of infertility and its cause, live birth, abortion, anti-mullerian hormone (AMH), level, IVF cycle characteristics, and the leading results were collected.

### Statistical analysis

Statistical data analysis was done using SPSS version 21 (IBM Corp., Armonk, NY, USA). First, we checked the normality of the variables using the Kolmogorov-Smirnov test. Then, an independent test was used to compare the significance of the variations among two independent groups, such as transferred embryo numbers. The Chi-square test (X^2^) was applied to test categorical variables such as gender and type of fertility. P-value<0.05 was considered statistically significant.

## RESULTS

**Figure 1:**
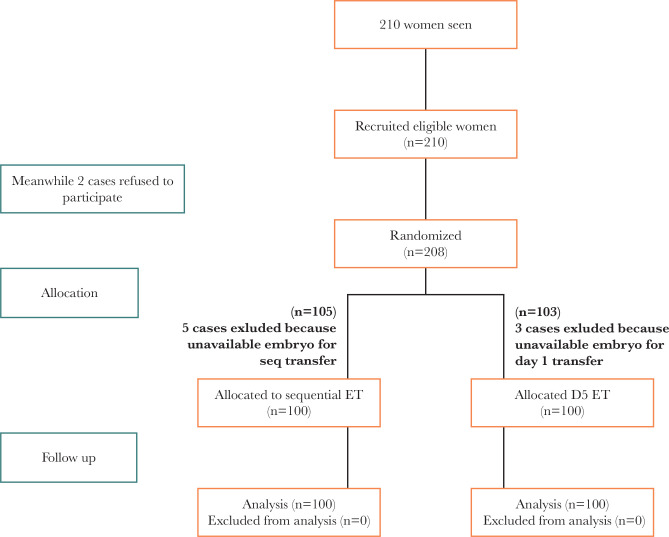
Flow diagram showing study course.

Out of 210 patients carrying out intracytoplasmic sperm injection (ICSI) who met the inclusion criteria, two participants in group one and two in group 2 were removed from the research for refusing to participate. Subsequently, 208 individuals were randomized in this research into two groups. Moreover, eight individuals were removed from both groups due to the lack of available embryos after thawing for transportation on day 3/day 5, or day 5. Finally, 200 individuals completed the study. The flowchart of the patients present in the research can be seen in Figure 1.

As presented in Table 1, there were no significant differences in patient characteristics among both groups in respect of age at the beginning of stimulation, BMI, time of infertility, the class of infertility, cause of infertility, AMH, baseline follicle-simulating hormone (FSH), sperm count, and morphology.

**Table 1:** Baseline characteristics of patients.

Variables	Intervention (n=100)	Control (n=100)	P-value
Age (year) (mean±SD)	35.06±4.33	33.90±4.00	0.051
BMI (mean±SD)	25.95±3.75	25.83±3.01	0.80
Infertility duration (years) (mean±SD)	6.92±2.40	7.48±2.13	0.082
Baseline FSH (mean±SD)	6.23±1.68	6.28±1.82	0.853
AMH (mean±SD)	2.01±1.76	2.28±1.34	0.226
Infertility type			
**Primary**	90 (90)	82 (82)	0.103
**Secondary**	10 (10)	18 (18)	
Cause of Infertility (n, %)			
**Male**	17 (17)	11 (11)	0.194
**Female**	29 (29)	40 (40)	
**Unknown**	54 (54)	49 (49)	
Sperm count (mean±SD)	27.23±10.17	31.21±16.05	0.038
Sperm morphology (mean±SD)	2.26±0.46	2.17±0.72	0.297

There were no statistically significant variations among the groups concerning the number of oocytes recovered (Table 2). A higher implantation rate was seen in the study group, but it was not statistically significant. The clinical pregnancy rate (per patient) was considerably greater in the sequential ET group than in the control group (P-value=0.004). Despite more transferred embryos in the study group, we did not have significantly different higher twin pregnancy and early pregnancy loss in the study group.

**Table 2:** Clinical variables and outcomes in patients.

Variables	Intervention (n=100)	Control (n=100)	P-value
**Number of oocytes retrieved (mean±SD)**	16.72±9.53	14.19±8.96	0.275
**Number of transferred embryo (mean±SD)**	3.34±0.699	3.16±0.801	0.014
**Implantation rate**	26 (26)	14 (14)	0.644
**Clinical pregnancy (n, %)**	40 (40)	19 (19)	0.004
**Twin pregnancy (n, %)**	12 (12)	9 (9)	0.342
**Abortion 2 (n, %)**	4 (4)	3 (3)	0.508

## DISCUSSION

Repeated implantation failure is often the cause of conditions associated with endometrial receptivity and embryo quality [5, 18, 19]. The most crucial step for successful IVF is transferring the best embryo exactly during the implantation window. However, it is not always easy to find the optimal time of endometrial receptivity.

Successful implantation involves complex crosstalk between the endometrium and the blastocyst. The presence of an embryo within the uterus stimulates the endometrium to produce many factors helping the embryo development and differentiating the endometrium to a receptive state [20]. These factors, like proteins that enhance growth, hormones, prostaglandins, adhesion molecules, and the extracellular matrix (ECM), can synchronize the embryo's growth to the blastocyst stage and differentiate the uterus from the receptive state [18, 21].

The idea of the sequential transfer comes from the concept that stimulation of endometrium earlier by a day 3 embryo may increase its' receptivity for a day 5 embryo. Furthermore, transferring embryos with a 2-day interval may also increase the probability of synchronizing different-stage embryos on the limited implantation window. Wakuda et al. showed that intraoviductal embryo in mice applies biological influences through the transfer of a message to epithelium and stroma of the endometrium, hence improving endometrial receptivity to the embryo and enhancing the degree of implantation [22].

The present research demonstrated that sequential embryo transportation on day 3 (cleavage ET)/day 5 (blastocyst ET) is related to significantly greater pregnancy rates than day 5 embryo transfer, consistent with some studies' findings. Hamdy and Deif showed that the sequential transfer of recovered oocytes in patients with enough oocytes on days 3 and 5 could be related to greater embryo implantation and clinical pregnancy frequency [23]. Also, Fang et al., in a retrospective case-control study, showed that day two and day 3 sequential transfer might improve pregnancy frequency in women with frequent IVF-embryo transfer failure [24].

Additionally, Loutradis et al. demonstrated that the greater pregnancy frequency with the double transfer method was an additional outcome success instead of day 4 [25]. Nevertheless, many authors fail to reveal any considerable enhancements in pregnancy frequency following the application of this method. Shahrokh Tehraninejada et al., and Al-Hasani et al., did not show a considerable difference in pregnancy frequency among the groups with seconder transfer [12, 14].

Machtinger et al. recommended that greater pregnancy rates can be linked to more embryos transported or to the method itself, which comprises 2 transfer processes.

They also revealed that multiple pregnancies were considerably more frequent in females undertaking sequential transfer, and the number of embryos transferred should be restricted to stop multifetal gestations [26]. Our study did not show a higher multiple pregnancy rate despite more transferred embryos in the sequential transfer group. Also, we did not find any substantial difference in the abortion rate among both groups.

Nevertheless, it is imperative to understand that the sequential method is appropriate for the normal responder individuals, who have enough quality embryos to transfer on the days of transfer together, and hence not appropriate for poor responders. So, these theories require prospective authentication in large-scale randomized trial studies with acceptable sample sizes. However, pending pregnancies were not assessed in this research, which was another limitation.

## CONCLUSION

Based on our study, sequential embryo transfer on day 3 and day 5 may enhance the clinical pregnancy frequency compared to the conventional day 5 embryo transfer in patients. Sequential embryo transfer could be considered in cases with a known past case of repeated IVF-ET failures with adequate good-quality embryos. More evidence from randomized clinical trials is needed.

## Personal thanks

We appreciate our colleagues at Avicenna Research Institute for their active contribution to this study. Also, we appreciate all cooperative patients participating in this clinical trial. Furthermore, we acknowledge all staffs who worked at Avicenna Fertility Center of Tehran for their great help in performing this study. Besides, Bahman Infertility and the staff helping to perform the study are gratefully acknowledged.
